# Editorial: The Red Cell Life-Cycle From Erythropoiesis to Clearance

**DOI:** 10.3389/fphys.2018.01537

**Published:** 2018-11-08

**Authors:** Lars Kaestner, Anna Bogdanova

**Affiliations:** ^1^Theoretical Medicine and Biosciences and Experimental Physics, Saarland University, Saarbrücken, Germany; ^2^Red Blood Cell Research Group, Institute of Veterinary Physiology Vetsuisse Faculty and the Center for Integrative Human Physiology, University of Zürich, Zurich, Switzerland

**Keywords:** red blood cell, Erythropoiesis, calcium, turnover, stored blood

Most of the articles in this research topic focus on processes associated with the plasma membrane of reticulocytes and mature erythrocytes. Membranes of red blood cells (RBCs) do not only serve as a barrier between the cytosol and the plasma compartments. They are actively involved in volume regulation, metabolic control, sensing of environmental stimuli, and control of life span of the circulating RBCs. Dynamic changes occurring at the membrane level play a key role in transformation of reticulocytes to mature RBCs and in selection of RBCs for clearance. Receptors initiate the signaling cascades that control gene expression in erythroid precursor cells undergoing differentiation. Fine-tuning of mRNA translation in differentiating erythroblasts is further controlled by RNA binding proteins that respond to the availability of growth factors, nutrients, and iron (Moore and von Lindern). Interference with signaling processes in control of proliferation and apoptosis, e.g. by way of introducing mutations into Ras GTPase RASA3 results in bone marrow failure syndrome and severe anemia. Using mouse model of the disease oxidative stress and membrane fragmentation during the terminal stages erythroid differentiation were revealed as a cause of disease (Hartman et al.). Enucleation and maturation of reticulocytes is associated with major changes in RBC membrane lipid and protein composition and massive re-arrangements of the membrane cytoskeleton (Moras et al.; Minetti et al.; Ovchynnikova et al.). These changes result from complex sorting processes in which some proteins are actively removed from the membrane surface whereas others are retained and even enriched if normalized to the membrane surface (Minetti et al.). Understanding of the mechanisms underlying reticulocytes production, release, and their maturation is a key to successful production of RBCs from stem cells in a bioreactor in the future (Ovchynnikova et al.).

Processes of maturation and aging of circulating RBCs are covered by several contributions to this volume. In nucleated RBCs of rainbow trout aging and senescence is associated with massive changes in DNA transcription along with loss of membrane surface and increase in density (Götting and Nikinmaa). Of importance, most of the age-dependent changes in transcriptome were reported for the genes involved in sensing stress (e.g., beta adrenoreceptors), ion transport and volume regulation (e.g., Na^+^/H^+^ exchanger) and in genes coding for cytoskeletal proteins (Götting and Nikinmaa). In human RBCs maturation and senescence is associated with decrease in deformability (Huisjes et al.). The spleen performs a selection for the least deformable cells that are not able to pass through the splenic sinuses, removing them from the circulation defining thereby RBC lifespan. Mechanical stress, the cells are exposed to when squeezing through the narrow capillaries, is sensed by mechano-sensitive ion channels that open upon mechanic stimulation and allow a short bout of Ca^2+^ entry (Danielczok et al.) that is followed by activation of Ca^2+^ pumps and rapid restoration of gradients (Kaestner et al.). Extreme sensitivity of RBCs to mechanical stress is almost never considered when designing experiments with these seemingly robust cells. In their study Wiegemann et al. show that simple pipetting or centrifugation may result in damage and loss of most unstable RBCs selecting more stable ones for further experiments.

Reduced oxygen availability (hypoxia) triggers adaptive responses in all cells and tissues including RBCs. In RBCs acute decrease in oxygenation is associated with a switch in metabolism favoring pentosophosphate pathway over glycolysis, increase in deformability and ATP release from RBCs of healthy humans (Grygorczyk and Orlov). Longer exposures of lowlanders to hypoxia triggers increase in RBC mass and the consequent upregulation in blood viscosity. RBCs newly produced at high altitude (neocytes) are most likely the first to be destroyed within the first days after descent from the mountains, the effect known as neocytolysis. The present state of research, the controversies and discussions of the neocytolysis concept are summarized in a review of Mairbäurl.

Extracoporeal circulation during open heart surgery represents one more stress condition causing RBC damage and hemolysis due to extensive mechanical abuse. Improvement of RBC membrane deformability and stability as well as reduction in oxidative stress by means of low-level light treatment supports RBCs and reduces hemolytic activity (Walski et al.). Stored RBCs also undergo premature removal that is driven by reversible and irreversible lesion formation. Whereas, membrane loss is associated with irreversible damage, metabolic depletion is a major contributor to the reversible lesion formation (Barshtein et al.). Membrane loss and the underlying alterations in cytoskeletal structure occurring during storage are making the cells less deformable. Mechanisms of microvesicle generation and their protein composition are intensively explored as they may provide insights into the such co-morbidities observed in patients with hereditary hemolytic anemias as thrombosis and autoimmune reaction (Leal et al.). Protein composition of vesicles produced during storage has been studied by Prudent et al. and provides further evidence that shedding of vesicle enriched with flotillin-2—band 3 complexes as well as α-adducin occurs during RBC storage. Loss of membrane or dehydration and the resulting decrease in deformability has multiple consequences. This important parameter may now be quantified and used as an indicator of severity of hereditary hemolytic anemias (Huisjes et al.). Recently introduced Laser Optical Rotational Red Cell Analyzer is becoming more and more abundant as a tool to assess deformability. Diagnostic power of this method for diagnosing rare hereditary anemias is discussed by Zaninoni et al. two processes converging and causing decrease in RBC deformability in senescent RBCs of healthy subjects include membrane loss and redistribution of inorganic cations and water. Loss of transmembrane gradients of Na^+^, K^+^, and Ca^2+^ in RBCs is known to be a common feature of several hereditary hemolytic anemias, often described as diseases of RBC volume regulation (Gallagher, 2013). Basic understanding of the molecular players in this dysregulation could result in new symptomatic therapies for this group of patients. In their review Flatt and Bruce give a detailed update on defects of cation permeability of RBC membranes in patients with hereditary stomatocytosis.

Free pseudo-steady state Ca^2+^ is maintained in the RBCs' cytosol at the level of approximately 60 nM (Tiffert et al., 2003) whereas plasma levels are up to 1.5 mM building up a gradient of more than four orders of magnitude. This gradient is supported by a low permeability of the plasma membrane of RBCs to Ca^2+^ and effective extrusion by the plasma membrane Ca^2+^ ATPases. Transient bouts of Ca^2+^ uptake via several types of Ca^2+^-permeable ion channels (Kaestner et al., accepted) sensitive to mechanical and chemical stimulation and changes in transmembrane potential are used for an extensive array of Ca^2+^-driven signaling in RBC (Danielczok et al.; Hertz et al.; Kaestner et al.). The role Ca^2+^ ions play in regulation of RBC life-span *in vivo* is discussed in several reviews in this volume (Lew and Tiffert; Romero and Hernandez-Chinea; Barshtein et al.).

Although, this research topic provides a significant contribution to our understanding of the RBC life-cycle, it is only one milestone on the way to go. We are delighted that in the frame of ‘Frontiers in Red Cell Physiology, new, more specialized research topics are initiated. We believe “Membrane Processes in Erythroid Development and Red Cell Life Time,” “Pathophysiology of Rare Hemolytic Anemias” and “Time Domains of Hypoxia Adaptation: Evolutionary Insights and Applications” and further research topics to come will facilitate RBC research and keep us updated.

## Author contributions

Both authors listed have made a substantial, direct and intellectual contribution to the work, and approved it for publication.

### Conflict of interest statement

The authors declare that the research was conducted in the absence of any commercial or financial relationships that could be construed as a potential conflict of interest.

## References

[B1] Gallagher P. G. (2013). Disorders of red cell volume regulation. Curr. Opin. Hematol. 20, 201–207. 10.1097/MOH.0b013e32835f6870 23519154

[B2] Kaestner L. Bogdanova A. Egee S. (accepted). Calcium channels and calcium-regulated channels in human red blood cells. Adv. Exp. Med. Biol. 10.1007/978-3-030-12457-1_25 31646528

[B3] Tiffert T. Bookchin R. M. Lew V. L. (2003). “Calcium Homeostasis in normal and abnormal human red cells,” in eds Bernhardt I. Ellory C. (Heidelberg : Springer Verlag), 373–405.

